# 573 The Impact of Burn Injuries on Indigenous Populations

**DOI:** 10.1093/jbcr/irad045.168

**Published:** 2023-05-15

**Authors:** Vincent Gabriel, Leah Verburg, Graham McCaffrey

**Affiliations:** Calgary Firefighters Burn Treatment Centre, Calgary, Alberta; University of Calgary, Calgary, Alberta; Faculty of Nursing, University of Calgary, Calgary, Alberta; Calgary Firefighters Burn Treatment Centre, Calgary, Alberta; University of Calgary, Calgary, Alberta; Faculty of Nursing, University of Calgary, Calgary, Alberta; Calgary Firefighters Burn Treatment Centre, Calgary, Alberta; University of Calgary, Calgary, Alberta; Faculty of Nursing, University of Calgary, Calgary, Alberta

## Abstract

**Introduction:**

Racial and ethnic minorities experience disparities in prevention and treatment of burn injury. However, research on burn injuries in Indigenous populations is limited. This review summarizes literature on burn injuries in Indigenous populations that require consideration due to systemic racism, intergenerational trauma, and health disparities to inform new research.

**Methods:**

A search was conducted in CINAHL, Ovid MEDLINE, PSYCinfo, and SocINDEX. for “burn OR scars OR scald OR deformity OR disfigurement” and “Aboriginal OR Indigenous OR First Nation OR American Indian OR Maori OR Native OR Torres Strait Islander OR Amerindian OR Inuit OR Metis OR Pacific Islander”. Search criteria were set by the authors with librarian assistance. Inclusion 1) peer-reviewed studies of burns in Indigenous persons 2) in English. Exclusion 1) no data specific to Indigenous burns 2) not peer-reviewed 3) not in full text 4) protocol publications. Selection was performed by a member of the research team and reviewed by the co-authors.

**Results:**

The search identified 1,091 studies with 51 for review. Fifteen were excluded. Three were editorials or letters. Five were not full text. One was a protocol only, and one did not reference Indigenous persons. Two reported accident rates in Indigenous populations but not burns data. Two excluded articles did not report burns data in the Indigenous population. One was excluded as the conclusions were not supported by the data. Included publications were published between 1987 and 2021 with 24 between 2011 - 2021. Indigenous populations have a rate of burn injury up to two times of the general population in the US, Canada, New Zealand, and Australia. Indigenous people suffered more injuries from flame and inhalation. Burns in Indigenous people are of greater total body surface area and deeper. Indigenous persons had longer lengths of stay, more complications, and need for additional surgeries. The Indigenous population in the US is associated with more hypertrophic scarring. Indigenous patients in Australia struggle with a lack of culturally safe communication and support for aftercare.

**Conclusions:**

Current literature is limited specific to burns in Indigenous populations. Racial disparities exist in burn injury incidence and outcomes for Indigenous persons. Indigenous populations suffer burn injuries, have more complications, and longer lengths of stay in hospital. Current care practices lack cultural safety considerations.

**Applicability of Research to Practice:**

Qualitative research in this area will help providers better understand the experiences of Indigenous burn patients to develop more culturally competent care. We are currently developing a study using qualitative hermeneutic methodology to learn about the experiences of Indigenous burn survivors’ injuries, recovery, and social reintegration.

